# Enhancing laying performance and immunity via probiotic and vitamin additives during induced molting

**DOI:** 10.3389/fvets.2024.1387877

**Published:** 2024-09-23

**Authors:** Chunyang Wang, Sa Xiao, Zengqi Yang

**Affiliations:** College of Veterinary Medicine, Northwest A&F University, Yangling, China

**Keywords:** probiotic and vitamin additives, induced molting, laying performance, immunity, laying hen

## Abstract

**Introduction:**

Molting is induced in commercial laying hens to rejuvenate the reproductive system and increase egg production. However, this process causes stress and reduces bird health and performance.

**Methods:**

The experiment was conducted to study the effect of multi probiotics and vitamin additives on induced molting in 240 ISA Brown hens. Hens were randomly divided into four groups receiving probiotic and vitamin additives (I–IV) during different period of molting. During the whole molting process, the laying performance indexes such as egg laying rate, egg quality, ovary weight and oviduct lengths were measured, and the spleen index, serum immunoglobulin, immune response of NDV and AIV vaccine were monitored.

**Results:**

Molted hens resumed 50% egg production in just 37 days, with 1.62% mortality. Egg quality such as egg weight, yolk color, Haugh unit, eggshell strength and protein height were significantly improved. After the second production peak, the reproductive organs and immune organs returned to normal, and the immune antibody titer of NDV vaccine increased significantly.

**Discussion:**

Molting with probiotic and vitamin additives improve the laying performance and egg quality, reduce mortality, significantly improve immune function and vaccine titer, and help to enhance disease resistance and maintain production performance of aged laying hens.

## 1 Introduction

Induced molting is a well-established technique widely used in the commercial egg industry across countries. Although molting raises animal welfare concerns, it rejuvenates flocks and extends the productive lifespan of laying hens by spurring reproductive system changes (1–3). Induced molting provides substantial benefits including increased egg production, improved egg quality, lower feed and production costs, and overall savings and revenue growth for large egg producers (4). However, conventional forced molting methods like grouping and culling hens have drawbacks like high mortality, prolonged molting periods, and limited production gains. Exploring new, effective molting techniques would also benefit the egg industry (5).

Supplementing laying hen diets with probiotics and vitamins enhances performance parameters including egg production, quality, ovarian function and stress reduction. Some studies report that probiotic supplementation significantly increases egg production and average weekly egg weight in late phase laying hens (6–9). Diets with multi probiotics increased egg production, weight, eggshell thickness, yolk color and Haugh units in laying hens (8, 10, 11). Probiotics also improved ovarian weight and ovarian function by increasing ovarian development-related genes like StAR, BMP15 and FSHR, ER gene expressions (9).

Dietary supplementation with vitamins A, B, C, D and E, individually and in combination, enhances egg production, quality, and immune response in laying hens. Elevating vitamin levels in aged hens’ diets improve production and egg quality (12). Vitamin E mitigates molting’s negative impacts on egg production and markedly increases Haugh units (13, 14).

A variety of probiotics and vitamins contribute to the enhancement of immune function. Probiotics can improve the intestinal microecological balance, increase the digestion and absorption rate of nutrients, and then enhance the production performance and immunity of laying hens (9). Addition of *Clostridium butyricum* and *Brevibacillus* increased significantly the level of IgM, IgA and immune organ index (7). Dietary supplementation with *Clostridium butyricum* increased levels of IgG and Spleen index (*p* < 0.05) (15). Furthermore, vitamin A and E supplementation had a significant effect on NDV or AIV antibody (16, 17). High level of water-soluble vitamins and vitamin E notably increased secretory IgA concentrations (12, 18). VD_3_ supplementation also reduced the increase of IgM level induced by LPS and stress (19).

Adjusting feed ratios and supplementing with vitamins, minerals and probiotics greatly affects induced molting efficacy (20). Since multi-probiotic and vitamin diets enhance egg production and ovarian function, we hypothesized similar effects may occur when supplementing replacement laying hens. There are limited studies on the application of a variety of probiotics and vitamin additives to induce molting, the purpose of this study is to explore the effects of those additives on laying performance, egg quality and immune function during induced molting, and promoting poultry scientific research and the application of new induced molting technology.

## 2 Materials and methods

### 2.1 Experimental animals

A total of 240,377-day-old ISA Brown laying hens were obtained from the Animal Experimental Centre of Northwest A&F University for the induced molting experiments. Another 39,982 laying hens from the same flock were molted at the same time to calculate the egg production rate and mortality rate, as well as to eliminate errors caused by the limited number of animals studied.

### 2.2 Experimental design and diets

The 240 hens were divided into four groups based on the phases of induced molting. Group I: Control group, non-molting. Group II: Sampled on day 11 of molting, during the feed restriction period with 0 egg production. Group III: Sampled on day 34 of molting, 25.53% egg production in the second laying cycle. Group IV: Sampled on day 49 of molting, peak 80.41% egg production in the second laying cycle. To ensure that all hens were the same age at the time of sampling at different molting stages, the groups started induced molting at different times, thus eliminating the interference of age variations on differences in physiological indices. Diets followed national standards for laying hens (GB/T 5916–2020). Table 1 shows diet composition and nutrition levels.

**Table 1:** Composition and nutritional content of basal diets.

Composition, %	Grower, pre-laying & laying feed content, %			Nutrient content	Grower, pre-laying & laying feed content, %		
Corn	65.0	65.0	57.4	Crude protein, %	15.5	17.0	17.5
Soybean meal	16.4	16.4	29.0	AME, MJ/kg^3^	2.8	2.75	2.7
Stone powder	6.0	6.0	3.81	Calcium, %	0.8	2.0	3.5
Wheat bran	7.0	7.0	3.17	Available phosphorus, %	0.60	0.55	0.60
Bone meal	1.5	1.5	2.5	Methionine, %	0.20	0.30	0.32
Shellfish powder	3.5	3.5	3.5	Lysine, %	0.45	0.60	0.65
Salt	0.3	0.3	0.37	Crude fiber, %	8.0	7.0	7.0
DL-Methionine	0.08	0.08	0.12	Crude Ash, %	10.0	13.0	15.0
Vitamin premix^1^	0.02	0.02	0.025	Moisture, %	12.0	12.0	12.0
Mineral premix^2^	0.1	0.1	0.1				
Total	100.00						

^1^Each kilogram of premix contains: Vitamin A 150,000 IU, Vitamin D_3_ 20,000 IU, Vitamin E 1,000 IU, Vitamin K 55 mg, Vitamin B_1_ 15 mg, Vitamin B_2_ 8 mg, Vitamin B_6_ 4 mg, Vitamin B_12_ 0.4 mg, Niacin 30 mg, Pantothenic acid 0.55 mg, Folic acid 0.4 mg, Biotin 0.16 mg, Choline chloride 450 mg.

^2^Copper 12 mg, Iron 120 mg, Zinc 100 mg, Manganese 50 mg, Iodine 20 mg, Selenium 0.4 mg.

^3^Metabolizable energy and nutrient content are calculated values.

### 2.3 Pre-experimental management

Unlike conventional methods, pre-molt removal of sick, weak, thin, obese, or depressed hens was not required. One week before molting, the poultry undergo Newcastle disease (ND) antibody testing. The henhouse and surroundings are thoroughly disinfected, with weekly sprinkling disinfection performed subsequently. Also, 1% limestone was added to feed 1 week before to reduce eggshell breakage and calcium depletion early in molting.

### 2.4 Experimental management

The patented “Farming Method for Repeated Molting of Laying Hens” developed by the Yangling Hongyan Molting Research Institute is employed to induce molting while adhering to strict procedural management in Table 2 (21). Different nutritional feeds and specialized additives were provided based on specific molting phases. These included various probiotics (*Bacillus subtilis*, *Lactobacillus plantarum*, *Lactobacillus acidophilus*, *Saccharomyces cerevisiae*), vitamins (vitamin A, B, C, D and E), Glucose, Niacin and Tryptophan, prepared as 6 different additive formulations used at different stages to provide targeted effects (Table 3). Housing temperature was maintained at 15 ~ 25°C during molting to optimize efficiency and minimize mortality. Throughout the experimental, eggs are collected at 09:00 and 14:00 daily, with artificial collection occurring toward the end of the laying cycle. Daily flock health and mortality were recorded.

**Table 2:** Specific implementation process of induced molting in laying hens.

Period			Items	
	Stage	Days	Feed	Water, Daily photoperiod
Implementation period	1–7	7	Stone powder 50 g/hen	Water supply, 8 h
	8–9	2	Stone powder 20 g/hen	Water withdrawal, 3 h
	10	1	Feed restriction	Intermittent water supply, 8 h
	11–14	4	Feed restriction	Water supply, 8 h
Recovery period	15	1	Grower Feed, 30 g/hen	Water supply, 8.5 h
	16	1	Grower feed, 60 g//hen	Water supply, 9 h
	17	1	Grower feed, 75 g/hen	Water supply, 9.5 h
	18	1	Grower feed, 90 g/hen	Water supply, 10 h
	19	1	Grower feed, 110 g/hen	Water supply, 10.5 h
	20–23	4	Pre-laying feed, 120 g/hen	Water supply, 11 h
	24–29	6	Laying feed, 120 g/hen	Water supply, Daily increase 0.5 h–16 h/d
	30–34	5	Laying feed, 120 g/hen	
2nd laying period	35–44	10	Laying feed, 120 g/hen	Water supply, 16 h
	45–49	5	Laying feed, 120 g/hen	Water supply, 16 h
	49	94	Laying feed, 120 g/hen	Water supply, 16 h

**Table 3:** Multi probiotic and vitamin additives used in the induced molting.

Additive I	Additive II	Additive III	Additive IV	Additive V	Additive VI
*Bacillus subtilis*		*Bacillus subtilis*			
*Lactobacillus plantarum*		*Lactobacillus plantarum*			
	*Saccharomyces cerevisiae*	*Saccharomyces cerevisiae*			*Saccharomyces cerevisiae*
*Lactobacillus acidophilus*					
Vitamin A	Vitamin A		Vitamin A		Vitamin A
Vitamin D			Vitamin D		Vitamin D
Vitamin E	Vitamin E		Vitamin E		Vitamin E
	Vitamin C				Vitamin C
				Vitamin B_1_	
				Vitamin B_2_	
				Vitamin B_6_	
Glucose					
			Niacin		
			Tryptophan	Tryptophan	

### 2.5 Evaluation of performance and egg quality parameters

On the day before molting, as well as the 4th, 8th and 14th days of feed restriction, about 1% of randomly selected hens were weighed. During the feed restriction period and 19 days after feeding was resumed, the number of mortalities was recorded. The duration of egg production cessation following feed restriction, as well as the time of egg production resumption after feeding were both recorded. Throughout the experimental, the overall egg-laying rate was recorded.

Egg quality parameters were measured at six distinct times during the molting induction process: the day before molting (376-day-old), the second day of feed restriction (378-day-old), the 4th day of feed restriction (380-day-old), when the egg-laying rate reached 25.53% after feeding resumption (410-day-old), when the egg-laying rate reached 50.33% after feeding resumption (413-day-old), and when the egg-laying rate reached 80.41% after molting (425-day-old).

At each time point, 10 eggs were randomly selected for assessment of egg quality-related characteristics such as egg weight, albumen height, Haugh unit, yolk color, shell strength, shell thickness and egg shape index computation. The longitudinal and transverse egg diameters were measured using a Vernier caliper to calculate the egg shape index (longitudinal diameter/transverse diameter). Shell strength was determined using an automatic eggshell strength tester EFG-0503 (Robotmation, Japan), and shell thickness was determined using an eggshell thickness gauge ETG-1601A (Robotmation, Japan). Egg weight, yolk color, albumen height and Haugh unit were determined using an automatic egg quality analyzer EMT-5200 (Robotics, Japan).

### 2.6 Tissue sample collection and organ index determination

Tissue samples were collected on each experimental group. The length of the oviduct was measured, and the ovaries were weighed. The organ index was calculated using the formula: Organ Index (g/kg) = Organ Weight (g)/Body Weight (kg). Blood samples of 2 mL were collected from the wing’s brachial vein and placed in EDTA anticoagulant tubes. The samples were centrifuged at 3000 r/min for 10 min, then the supernatants were extracted and stored at −80°C before testing.

### 2.7 Determination of spleen index and immunoglobulin levels

Three laying hens were randomly selected from groups for weight measurement and dissected. Spleen was extracted, body fat was removed, body fluid was aspirated with filter paper and weighed. The spleen index = Fresh spleen weight (mg)/Body weight before slaughter (kg). Immunoglobulin A (IgA), immunoglobulin M (IgM) and immunoglobulin Y (IgY) levels in serum were determined by ELISA kits (BEIJING SINO-UK, China).

### 2.8 Measurement of the NDV and AIV HI antibody

After induced molt, 20 layers were selected from control group I and molting group IV. Control group and Molting group were vaccinated with NDV and AIV vaccines, respectively. Blood was collected at 7, 14, 21 and 28 days post-vaccination. Hemagglutination inhibition (HI) tests were used to determine NDV and AIV antibody titers.

### 2.9 Analytical statistics

The experimental data were analyzed using IBM SPSS Statistics 26. One-way analysis of variance (ANOVA) was performed, followed by Tukey’s *post hoc* test for multiple comparisons. GraphPad Prism 9.0 was used for data visualization, analysis of differences, and determination of statistical significance based on *p*-values. *p*-value less than 0.05 was considered statistically significant, while *p*-values greater than 0.05 indicated no statistically significant difference. The results were presented as the mean ± standard deviation.

## 3 Results

### 3.1 Multi probiotic and vitamin additives improve egg-laying rate in hens

**Figure 1:**
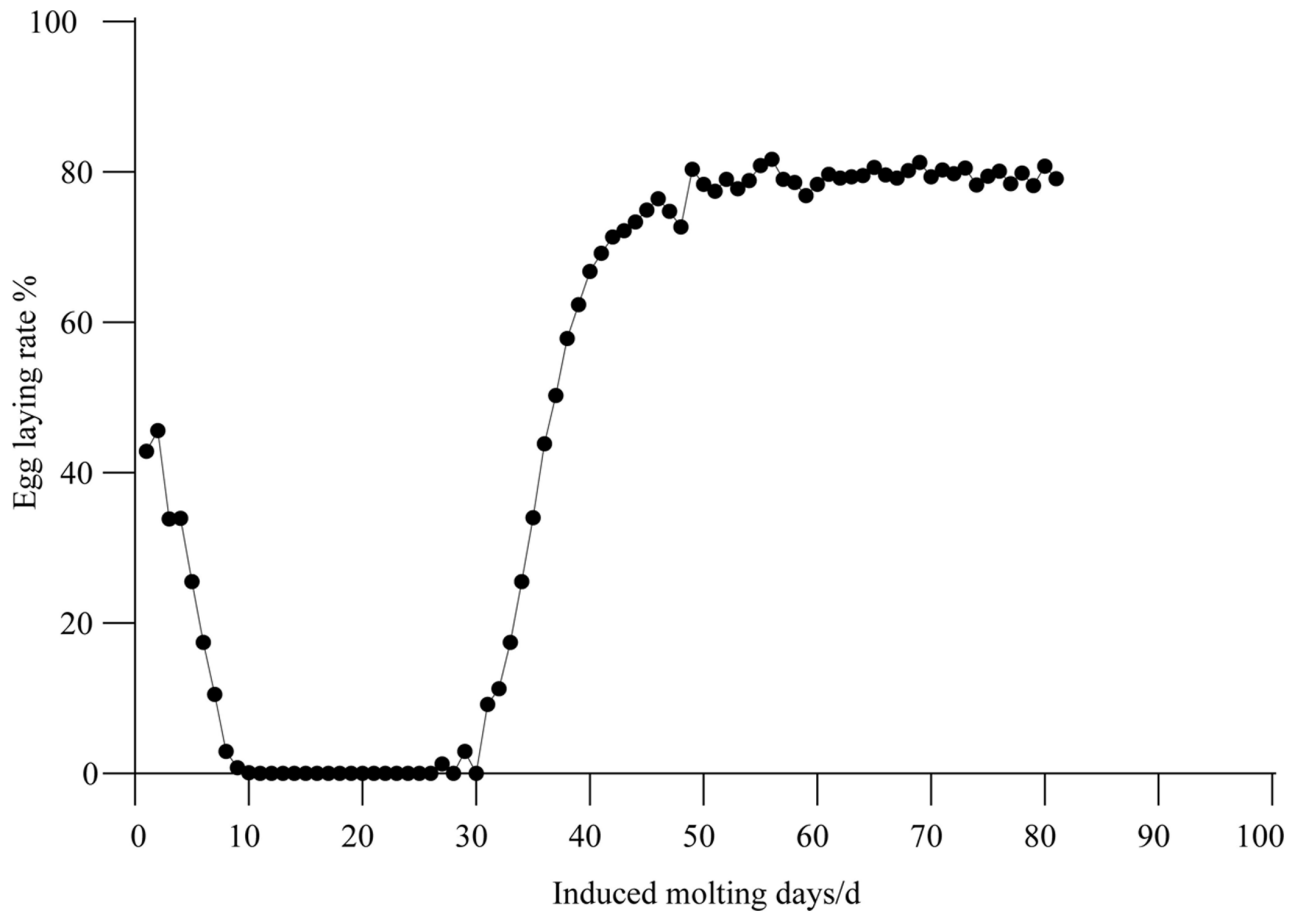
Alterations in egg production rate during the molting period.

Multi probiotics and vitamins supplements had a significant impact on egg laying rates in hens undergoing induced molting. Before molting, the baseline egg laying rate was 42.84%. During feed restriction, egg production progressively declined, reaching zero by day 11. However, following refeeding, the population’s laying rate exceeded 50% by day 37 and further increased to 80.41% by day 49. The population consistently maintained ≥75% laying rate for over 80 days (Figure 1).

### 3.2 Body weight declines during feed restriction period

Body weight (BW) changes were monitored during feed restriction. On the day before induced molting, 385 randomly selected hens averaged 2148.9 g. By day 14 of restriction, 361 randomly weighed hens averaged 1605.2 g, indicating over 25.30% weight loss. Gradually resumed feeding was decided based on flock condition (Table 4).

**Table 4:** Decrease in average BW during the restriction feeding period.

Restriction period	Number of weighed hens, hen	Average weight, g	Weight loss rate, %
Day before molting	48	2148.9	0
Day 4 of restriction	47	1858.8	13.50
Day 8 of restriction	46	1689.0	21.40
Day 14 of restriction	46	1605.2	25.30

### 3.3 Mortality rate fluctuations during induced molting

Mortality rates were monitored during feed restriction. Average daily mortality was calculated at 3-day intervals. Based on Table 5, 403 hens died during restriction, averaging 1.01% daily. Notably, average daily mortality was significantly higher on days 7 ~ 12 versus the first 6 days.

**Table 5:** Mortality rate during the restricted feeding period.

Days, d	Total culling count, hen	Total population, hen	Daily average mortality rate, %
1 ~ 3	43	39,967	0.11
4 ~ 6	67	39,892	0.17
7 ~ 9	107	39,789	0.27
10 ~ 12	103	39,698	0.26
13 ~ 15	83	39,609	0.21
Total	403	39,982	1.01

**Figure 2:**
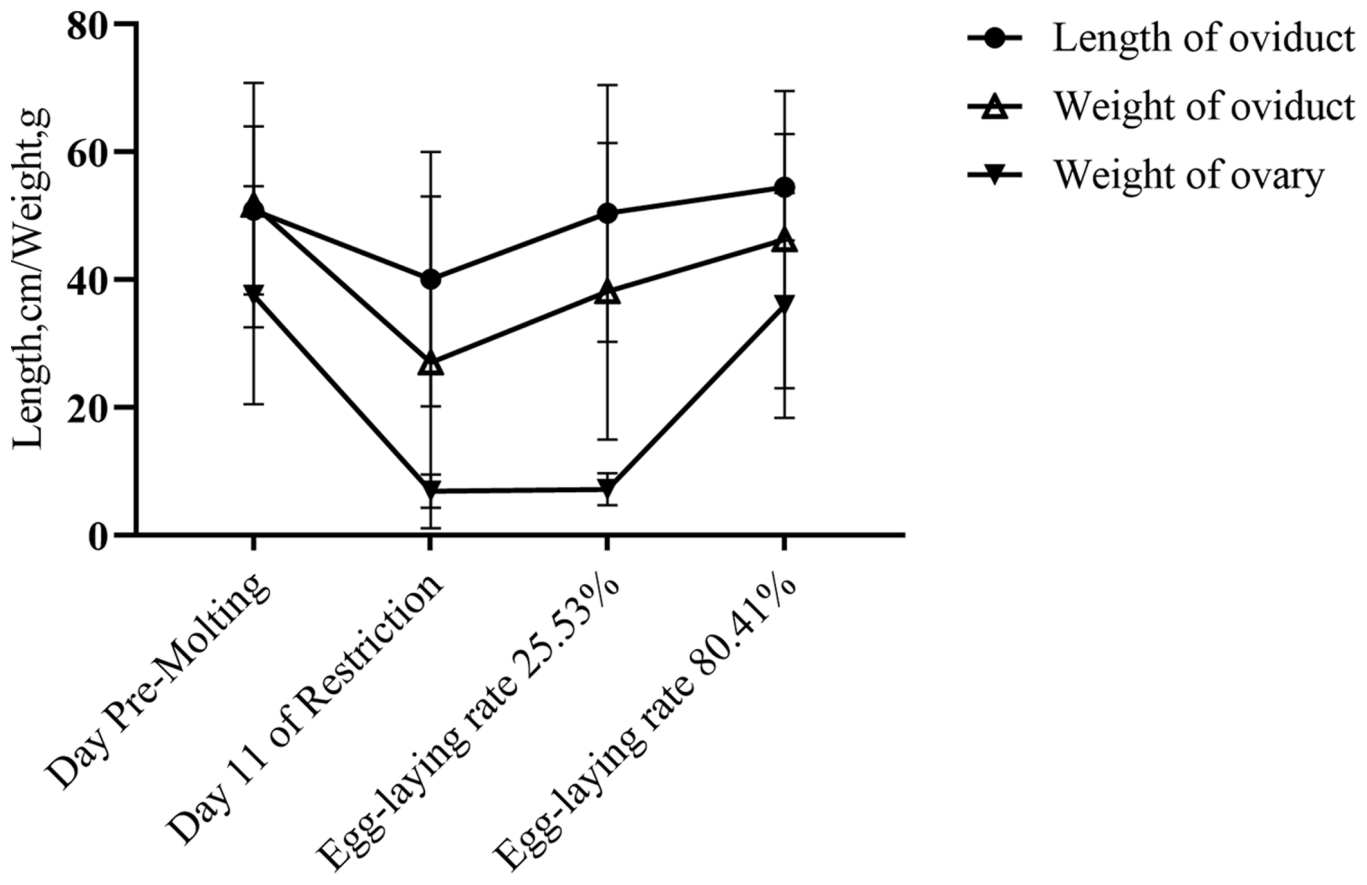
Alteration in reproductive organs during the induced molting.

After refeeding, average daily mortality was calculated at 3-day intervals until 50.33% egg laying rate. According to Table 6, 246 hens died in the first 22 refeeding days, averaging 0.61% daily. Significantly higher mortality occurred on the first 2 refeeding days versus days 3th to 22th.

**Table 6:** Daily average mortality rate during the resumption of feeding period.

Days, d	Total culling count, hen	Total population, hen	Daily average mortality rate, %
1 ~ 3	113	39,486	0.29
4 ~ 6	54	39,426	0.14
7 ~ 9	22	39,395	0.06
10 ~ 12	10	39,382	0.03
13 ~ 15	9	39,374	0.02
16 ~ 18	13	39,362	0.03
19 ~ 22	25	39,342	0.04
Total	246	39,982	0.61

### 3.4 Induced molting improves on egg quality

Egg quality was assessed at several timepoints during induced molting. As shown in Table 7, egg weight before molting and on feed restriction day 4 was lower than the period in 50.33 and 80.41% laying rates after refeeding (*p* < 0.05). However, there was no difference in egg weight between the 50.33 and 80.41% laying rates (*p* > 0.05). Egg shell strength initially decreased, then increased during molting, Egg shell strength initially declined, then increased during molting, eventually exceeding pre-molting levels. Egg weight, egg shell strength, albumen height and yolk color all increased relative to pre-molting levels.

**Table 7:** Variations in egg quality during induced molting using multi probiotic and vitamin additives.

Items	Day pre-molting	Day 2 of restriction	Day 4 of restriction	Egg-laying rate 25.53%	Egg-laying rate 50.33%	Egg-laying rate 80.41%	*p*-values
Egg weight, g	46.83 ± 5.13^b^	50.66 ± 7.43^b^	45.90 ± 5.26^b^	47.42 ± 3.79^b^	51.67 ± 4.80^ab^	56.41 ± 6.06^a^	<0.001
Eggshell thickness, mm	0.37 ± 0.04	0.37 ± 0.03	0.34 ± 0.04	0.37 ± 0.04	0.37 ± 0.04	0.37 ± 0.04	0.420
Egg shape index	1.31 ± 0.09	1.31 ± 0.08	1.26 ± 0.06	1.30 ± 0.06	1.29 ± 0.04	1.33 ± 0.05	0.277
Eggshell strength, N	28.15 ± 11.09	25.57 ± 11.51	25.33 ± 11.99	28.54 ± 8.27	30.46 ± 11.28	31.85 ± 10.67	0.723
Albumen height, mm	3.10 ± 0.66	3.23 ± 0.26	2.80 ± 0.46	4.14 ± 1.68	3.70 ± 1.30	3.60 ± 1.70	0.150
Egg yolk color	9.17 ± 0.99^b^	10.53 ± 1.82^ab^	9.73 ± 0.21^ab^	9.61 ± 1.13^ab^	10.64 ± 0.78^a^	9.44 ± 0.45^ab^	0.011
Haugh unit	52.70 ± 9.17^b^	72.20 ± 25.55^a^	52.87 ± 10.53^b^	60.80 ± 10.33^ab^	58.75 ± 12.75^ab^	48.65 ± 7.47^b^	0.007

^a^,^b^Different lowercase letters within the same row indicate significant differences (*p* < 0.05), while the same lowercase letter or no letter indicates no significant difference (*p* > 0.05).

### 3.5 Reproductive organs changes during induced molting

Changes in oviduct and ovary weight and length were monitored during induced molting (Figure 2). Both decreased then increased from baseline. On feed restriction day 11, oviduct length, weight and ovarian weight all decreased, although the differences were not statistically significant (*p* > 0.05). As laying rate recovered to 25.53% following refeeding, oviduct length and weight reverted to pre-molting levels. No significant ovary weight changes occurred.

At the 80.41% peak laying rate, no significant differences occurred in oviduct or ovary weight and length compared to pre-molting (*p* > 0.05). As indicated in Table 8, there were no significant between-group variations occurred in pre-slaughter weight, oviduct index, or ovary index (*p* > 0.05), indicating decreasing then increasing trends.

**Table 8:** Alteration in reproductive organ indices during induced molting.

Items	Day pre-molting	Day 11 of restriction	Egg-laying rate 25.53%	Egg-laying rate 80.41%	*p-*values
Pre-slaughter weight, kg	2.20 ± 0.05	1.85 ± 0.11	2.14 ± 0.14	1.86 ± 0.18	0.166
Oviduct index, ‰	28.45 ± 3.66	16.11 ± 7.17	21.22 ± 6.27	26.64 ± 5.60	0.461
Ovary index, ‰	17.22 ± 3.96	7.65 ± 4.45	3.30 ± 0.38	14.35 ± 6.30	0.151

### 3.6 Effects of induced molting on immune organs and immunoglobulin levels

**Figure 3:**
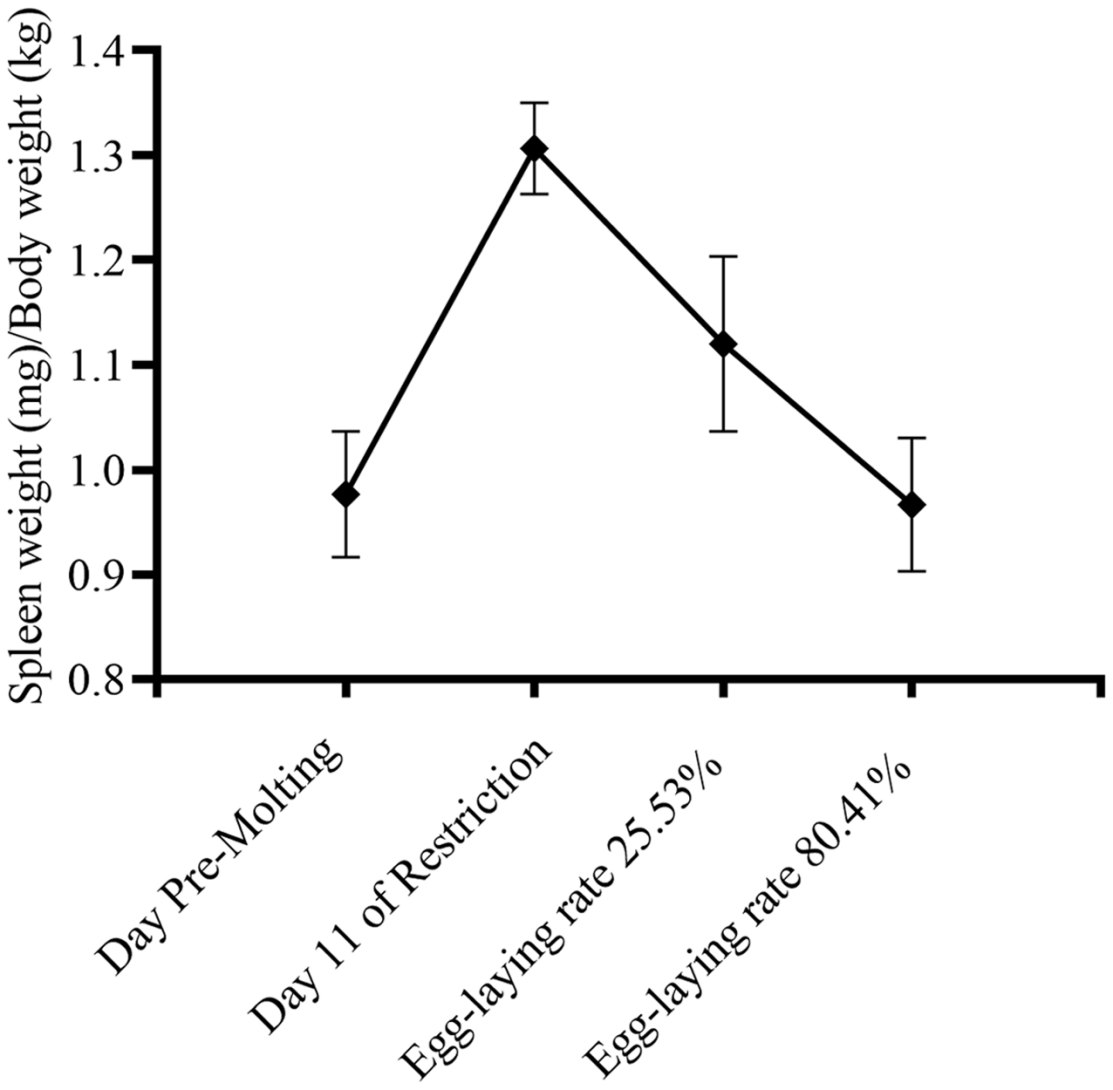
Alteration in spleen index during the induced molting.

**Figure 4:**
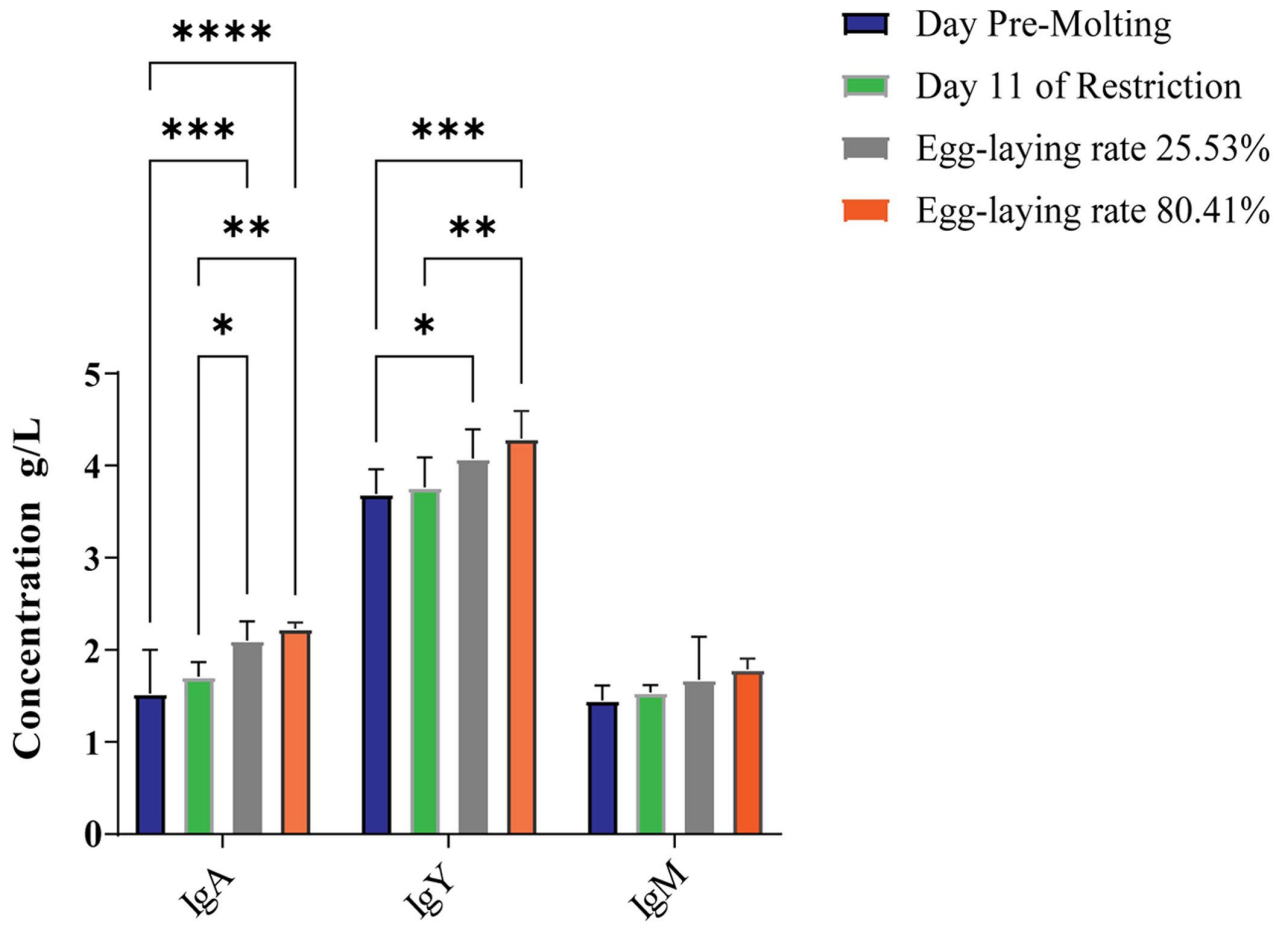
Effects on the immunoglobulin levels of laying hens during molting. * represents *p* < 0.05, ** represents *p* < 0.01, *** represents *p* < 0.001.

As seen in the Figure 3, the spleen index grew dramatically after 14 days of fasting, and with refeeding, it returned to pre-molting level. During induced molting, the contents of IgA, IgY and IgM change dynamically as Figure 4. IgA, IgY and IgM levels were higher post-molting than before. The levels of IgA and IgM in egg-laying peak post-molting were significantly greater than control group (*p* < 0.05), and IgY also increased.

**Figure 5:**
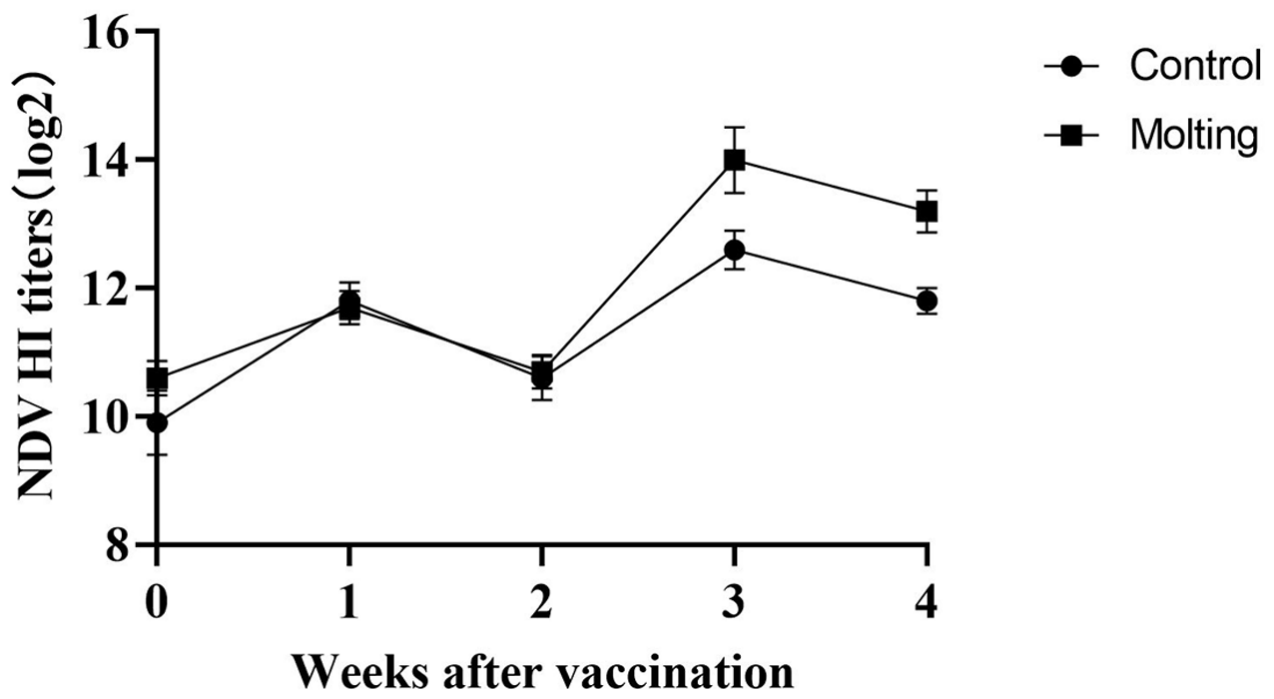
Effects on immune response to NDV vaccination post molting.

### 3.7 Effects of induced molting on immune response to NDV and AIV vaccination

**Figure 6:**
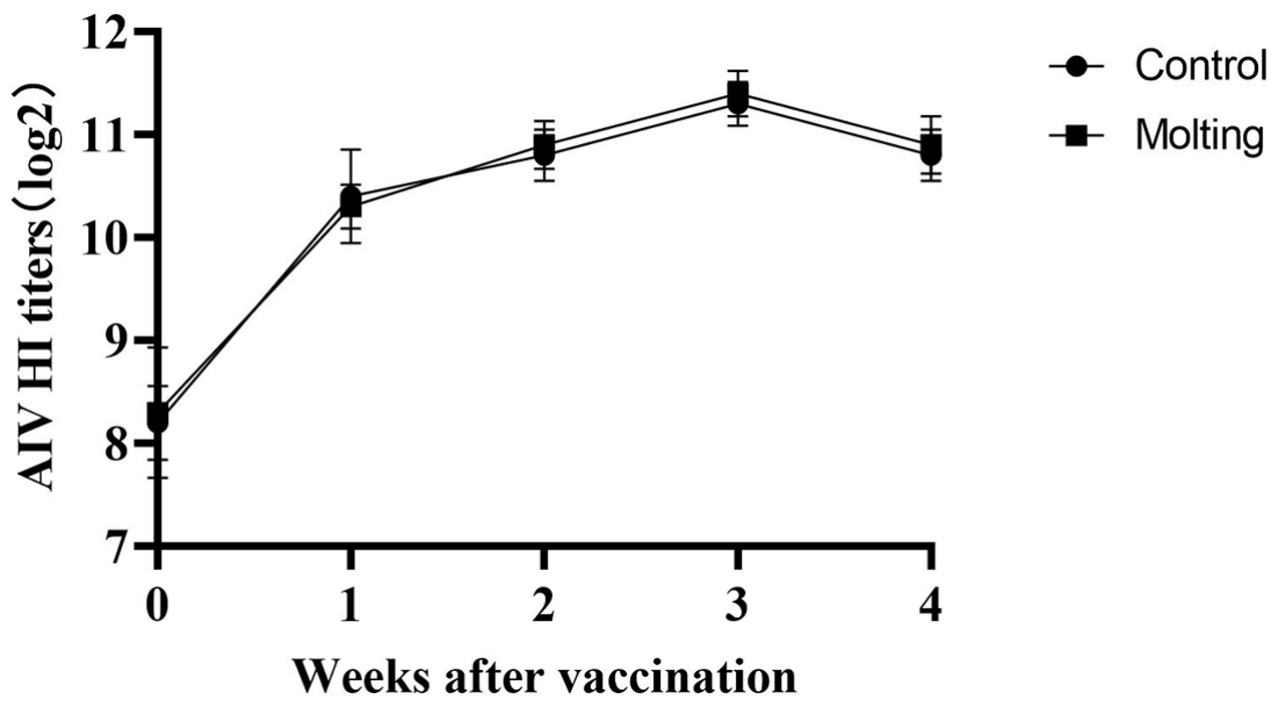
Effects on immune response to AIV vaccination post molting.

At 3, 4 weeks after NDV immunization, there were significant differences in HI antibody titers between the control and induced molting group (*p* < 0.05), with higher NDV antibody titers in the molted hens (Figure 5). However, there were no significant differences in HI antibody titers against AIV across the groups (*p* > 0.05). The results revealed that induced molting significantly raise the NDV antibody titers while simultaneously increasing the AIV antibody titers, although the effect was not significant (Figure 6).

## 4 Discussion

### 4.1 Probiotics and vitamins enhance post-molting laying rate

Previous studies show induced molting increase hen laying rates in the second egg cycle (1, 22, 23). Alodan et al. found molting elevated rates from 64 to 83% (24). A 7-day food restriction program also improved egg production (25). Wu et al. discovered that post-molting laying rates were 79.9 and 76.9% for fasting and low-salt programs, respectively (26). In this study, induced molting with multi probiotics and vitamins remarkably increased peak laying rate to 80.41% in the second cycle, 37% higher than pre-molting. This compensates for economic losses from higher costs and lower egg prices. Peak laying rate was maintained for 84 days after molting with a combination of probiotics and vitamins. This extended older hen usage and culling flexibility, enabling production adjustments based on market conditions to minimize farming business risks.

In this research, probiotics and vitamins were used to stimulate molting, resulting in 50% egg production 37 days after refeeding began. Typically, 7 ~ 9 weeks are needed to recover a laying rate of more than 50% after induced molting in healthy hens (27). Thus, probiotics and vitamins additives in induced molting resulted in increased second cycle laying rates, shorter molt time, lower feeding costs, and enhanced production efficiency.

### 4.2 Weight loss during the restricted dietary period

In addition to flock condition, weight loss rate during feed restriction serves as a quantitative indicator for refeeding timing. Successful molting generally results in a 25 ~ 30% loss. It has also been reported that optimal post-molt performance occurs around 27 ~ 31% loss (28). Maximum increase in post-molt egg production rate occurred at 30% of body weight loss (29). In this experiment, refeeding began at 25.30% weight loss based on evaluating overall health, weight loss rate, and mortality.

Previous studies show greater weight loss in feed withdrawal associated with higher second cycle laying rates, with a negative linear relationship (30, 31). This greater weight reduction may explain why the egg production is lower than predicted post-molt here. Controlling weight loss during feed restriction is crucial for optimizing molting process and the second laying cycle, enabling for effective hen health and productivity management while minimizing any potential negative impacts.

### 4.3 Reducing mortality rate during induced molting

Fasted molt mortality in the United States should keep below 1.2% with measures to minimize flock loss (1). Here, mortality was 1.01% during feed restriction and 1.62% overall, significantly lower than methods such as feed withdrawal (9.9%), high zinc feeding (5.5%), moderate-Low-Zn/Ca–P deficiencies (2.8 ~ 4.2%), low-sodium diet (3.4%) wheat middlings/corn diet (2.8%), and 10-day fasting followed by corn refeeding (1.39%) (32, 33). Mortality was relatively low in the first 7 restricted days, probable because to coarse stone powder, multiple probiotics and vitamins.

In the first 1 ~ 2 refeeding days, average daily mortality reached 0.29% but then decreased substantially, normalizing after 7 days. Abrupt refeeding may cause injurious feeding frenzy. Smaller, weaker hens may have limited access to feed or be trampled to death. Earlier feed preparation and later light exposure (15 ~ 30 min) may reduce mortality, but further researches are needed.

### 4.4 Induced molting improves egg quality

Induced molting significantly impacts late-stage layer egg quality. Some studies show higher second cycle egg weights versus non-molted hens (29). In this experiment, average egg weight post-molting increased 9.58–56.67 g. Increased eggshell strength reduces fractures and breakage, directly improving commercial egg output and revenues. Strength initially reduced, then increased throughout molting, eventually exceeding pre-molting values, consistent with prior studies (22). *Bacillus subtilis* or *Lactobacilli* additions have been shown in studies to increase eggshell strength, which is compatible with the probiotic benefits found here. Similarly, studies suggest that microbial additions boost protein production, whereas vitamins A, C and E considerably improve egg quality in layers (9, 12, 34–38).

This supports the use of multiprobiotics and vitamins to enhance quality during induced molting. Further research should optimize special probiotic strains, vitamin types and levels for induced molting protocols.

### 4.5 Alterations in reproductive organs through induced molting

Induced molting causes structural and functional changes in reproductive organs, affecting subsequent egg production and egg quality (39). After 11 days of feed restriction, oviduct and ovary weights significantly drop, ceasing egg production. This reproductive organ atrophy may be caused by inadequate energy intake during molting, resulting in nutrition conservation responses (40).

Different molting methods do not affect the difference in ovarian and oviduct weight (41). In the early refeeding phases, energy intake restores weight and repairs damage from fasting, while follicles start developing, and in the late stage of recovery, body weight and reproductive organs recovered (42). Following induced molting, oviduct length, weight, and ovary weight all returned to pre-molt values, indicating regained function and increased second cycle productivity.

### 4.6 Induced molting helps enhance immune function

Spleen index reflects the body’s nonspecific immune capabilities, as the spleen index increases, the immune function enhanced, which may be due to increased activity of immune cells in the spleen, resulting in an increase in spleen volume and weight (43). Earlier research found no significant differences in spleen index during molting (44). Our findings suggest that the spleen index increased significantly during fasting period, with no significant difference after refeeding. Fasting stress caused the multiplication of spleen immune cells to compensate for the body’s immunological function may be the reason.

Compound probiotics have been shown to increase the expression of immunity related genes (TLR2, IL-2) in broilers (45). Dastar et al. discovered that when Calcium, lactose and probiotic was added during molting, serum IgG and IgM levels were higher than full feed group, which suggests that probiotics and prebiotics have a strong potential to regulate immunological response (44). Another research discovered that the level of IgA, IgG and IgM was higher than pre-molt when it reached the second peak production (46). Fasting redistributes peripheral circulating immune cells, and fasting and a gradual feeding will reshape innate immune function (47). Early studies indicated that varied molting procedures had no adverse effects on antibody generation or immunological response (24).

Someone found that significantly higher level of serum IgG during forced molting than non-molting group (48). Our finding is in agreement with recent results that the IgA and IgM are much higher than that pre-molting, indicating that the induced molting operation was helpful to improve the body resistance.

The sixth day of fasting has been reported to enhance the immune response to various antigens, especially viral respiratory diseases, this positive immune enhancement is sustainable and does not diminish after refeeding (47). Berry and Alodan et al. found that molting does not affect the antibody titer against NDV and AIV in commercial layers (24, 39). Another study also found no change in NDV and AIV antibody titer in laying hens post molting (25). However, the latest study indicated molting significantly boosted NDV antibody titer (*p* < 0.05), but had no significant effect on AIV titers (49). During molting, the addition of 16% vitamin E and 16% vitamin C dramatically boosted the immunological response of NDV antibody (50). Similarly, the results showed that the antibody titer against NDV was greatly enhanced post-molting, whereas the antibody titer against AIV was not significantly increased, which could be due to the antigenicity of NDV is relatively stable, while AIV is easy to mutate, resulting in differences in the vaccine’s immune response to the two viruses. The changes in immune organs, serum immunoglobulin and vaccination antibodies demonstrated that induced molting updated the immune system to some extent, which was beneficial for disease prevention and management in large-scale laying hen breeding post molting.

## 5 Conclusion

Induced molting with multi-probiotics and vitamins improves production and egg quality, shortens the molting cycle, reduces the mortality rate of aged laying hens. Serum immunoglobulin and NDV vaccine antibody increased significantly after molting, which is helpful to enhance the innate and acquired immune response and resistance to disease. In general, using a variety of probiotics and vitamin additives during molting effectively improve the production performance and disease resistance of aged laying hens.

## Data availability statement

The raw data supporting the conclusions of this article will be made available by the authors, without undue reservation.

## Ethics statement

The animal study was approved by Animal Experimental Centre of Northwest A&F University. The study was conducted in accordance with the local legislation and institutional requirements.

## Author contributions

CW: Formal analysis, Investigation, Methodology, Writing – original draft. SX: Supervision, Writing – review & editing. ZY: Supervision, Writing – review & editing.
